# Integrating Hormonal, Environmental, and Genetic Cues in Strawberry Flowering

**DOI:** 10.3390/plants15213378

**Published:** 2026-11-01

**Authors:** Jiajie Yu, Xuemei Xia, Zhihan Niu, Ziwei Jia, Xiang Zhang, Xiaohong Li

**Affiliations:** School of Agriculture, Liaodong University, Dandong 118003, China; yujiajie@liaodongu.edu.cn (J.Y.); 13624156318@163.com (X.X.); n51nzh@163.com (Z.N.); 15142553449@163.com (Z.J.)

**Keywords:** strawberry, flowering, environmental regulation, phytohormone, molecular network

## Abstract

Strawberry is an important fruit crop around the globe. Its yield and quality are fundamentally and closely related to flowering. This review synthesizes current knowledge on the morphological, environmental, hormonal, and molecular regulation of strawberry floral development and the integration of these regulatory layers. Morphologically, strawberry flower differentiation develops from shoot apical meristem transition through sequential floral organ initiation, culminating in the unique pseudocarpic fruit structure. Environmentally, photoperiod, temperature, and light quality are the primary signals governing strawberry flowering. In seasonal-flowering genotypes, this module typically shifts the balance toward the TFL1–FD–14-3-3 repressor complex, suppressing floral identity genes and repressing flowering. In perpetual-flowering *F. vesca* genotypes, loss of functional *FvTFL1* shifts the balance toward the FT–FD–14-3-3 activator complex, promoting continuous flowering. In cultivated octoploid strawberry, the genetic basis of perpetual flowering is more complex and involves additional loci beyond *TFL1*. Hormonally, gibberellins, cytokinins, auxin, abscisic acid, ethylene, and jasmonic acid modulate the core photoperiodic regulation at multiple nodes. Molecularly, these signals converge on integrated networks in which the *CO–FT1–SOC1–TFL1* module plays a central role, auxin signaling directly activates floral meristem identity genes, and octoploid-specific regulatory features add further complexity. This review provides an integrated overview for future research reference and breeding of strawberry flowering.

## 1. Introduction

Strawberry is a globally cultivated fruit crop possessing significant economic and ecological value. It enjoys exceptional consumers’ preference in fruit markets, favored for its elegant fruit shape, delightful smell, superior taste, and dense nutritional content, including vitamin C, dietary fiber, folate, manganese, and anthocyanin [1]. The key to successful strawberry cultivation fundamentally depends on the normal and precise timing of flowering, which is a developmental process that determines fruit yield, quality, and the synchronization of production with market windows [2,3].

Strawberry flowering includes a continuum of developmental events, from the environmental induction of the shoot apical meristem (SAM) to the differentiation of floral organs and the subsequent development of the receptacle into the fleshy fruit [4,5,6]. This process is fine-tuned by the integration of external environmental signals, including photoperiod, temperature, and light quality, the foundation of which is an increasingly well-characterized molecular network. At the core of this network lies the *FvCO–FvFT1–FvSOC1–FvTFL1* regulatory module [7,8,9]. This module was first elucidated in the diploid woodland strawberry (*Fragaria vesca*) and subsequently extended to the cultivated octoploid species. More recent work has found that *FveFT2* functions as a non-photoperiodic florigen permitting short-day flowering, while *FveFT3* promotes plant branching and can increase fruit yield by 3.5-fold at the expense of stolons [10]. This underscores the intricate balance between sexual and asexual reproduction in this perennial species.

Despite the research frontiers, a comprehensive understanding of how environmental, hormonal, and genetic cues are integrated to regulate strawberry flowering remains worthy of discussion and exploration. The molecular basis of temperature sensing and signal transduction is not abundantly unveiled in cultivated strawberry, of which the octoploid genome and high heterozygosity introduce additional regulatory complexity [11]. Transcriptomic analyses have revealed that the plant hormone signal transduction pathway is the most enriched category among differentially expressed genes during early floral initiation [12]. It has been found that genes associated with auxin signaling, cytokinin receptors, and ethylene response factors are dynamically regulated across developmental stages [5,13]. Gibberellin (GA) application has been shown to delay floral transition through the coordinated regulation of photoperiod signaling and nitrogen metabolism, while cytokinins (CKs) are involved in flower and receptacle development [13,14]. These hormonal pathways do not operate independently, but converge on the core photoperiodic module, modulating the expression of floral integrators and meristem identity genes. However, the detailed mechanism of this convergence, particularly how auxin response factors and cytokinin signaling components integrate with the photoperiodic pathway, remains incompletely investigated.

This review aims to synthesize current knowledge on the morphological, environmental, hormonal, and molecular regulation of strawberry flower formation and the integration of these regulatory layers. We first establish the morphological and developmental framework of strawberry flower differentiation, including the distinctive features of its pseudocarpic fruit and the divergent developmental trajectories of seasonal-flowering (SF) and perpetual-flowering (PF) varieties. We then describe the environmental signals, including photoperiod, temperature, and light quality, which govern floral induction. The hormonal pathways that transduce these signals into developmental outputs are also introduced. The molecular networks that integrate hormonal, environmental, and genetic cues are subsequently analyzed, with attention to both the conserved core modules identified in diploid woodland strawberry (*Fragaria vesca*) and the cultivar-specific variations observed in octoploid strawberry (*Fragaria × ananassa*). Finally, we discuss current controversies, knowledge gaps, and future directions for research and breeding. By providing a comprehensive and integrative perspective, this review aims to advance both fundamental understanding of strawberry flowering biology and the development of strategies for optimizing flowering in cultivation.

We think it necessary to facilitate transparency regarding the strength and source of evidence supporting the major mechanistic claims discussed in this review. The supporting evidence for each claim, distinguishing between experimental validation in *F. vesca*, experimental validation in *F. × ananassa*, transcriptomic correlations, heterologous evidence from *Arabidopsis*, and proposed or inferred mechanisms, is summarized in the Supplementary file (Table S1).

## 2. Morphological and Developmental Framework of Strawberry Flower Formation

A clear morphological framework is essential for interpreting the environmental, hormonal, and molecular regulators that are involved in strawberry flowering. It is well worth illustrating given strawberry’s unique pseudocarpic fruit structure, derived from the floral receptacle, and the existence of both seasonal-flowering and perpetual-flowering varieties with markedly different developmental patterns. Accordingly, this section outlines the morphogenetic progression of flower differentiation, describes the distinctive features of strawberry floral development, and compares the two flowering habits, thus establishing the spatiotemporal context for the regulatory mechanisms examined in subsequent sections.

### 2.1. Morphogenesis of Strawberry Flower Differentiation

Strawberry flower differentiation represents a critical developmental transition during which the shoot apical meristem (SAM) shifts from vegetative to reproductive growth [15]. It ultimately generates floral organs that determine yield and fruit quality. This process is regulated by a complex of endogenous genetic profiles and environmental factors and has been most comprehensively characterized in the diploid woodland strawberry (*Fragaria vesca*).

In *F. vesca*, the vegetative SAM produces leaf primordia and lateral branches during the vegetative phase [16,17]. Upon floral induction, the SAM undergoes a morphological transformation, ceasing leaf differentiation and changing from a flattened shape to a domed configuration [18]. This dome-shaped structure becomes the inflorescence meristem, which subsequently terminates in a floral meristem (FM) [16]. The initiation of the inflorescence is first evident as a raising and enlargement of the apical meristem; floral initiation then proceeds in a centripetal manner, with sepals appearing first, followed by petals, stamens, and pistils [19].

Detailed developmental staging of *F. vesca* flower development has been established through histological sections and scanning electron microscopy [20]. This provides standardized models for morphological changes associated with flower and early fruit development. For cultivated strawberry, *Fragaria × ananassa* flower development has been divided into 16 stages according to landmark events, which are similar to those documented for *F. vesca* but include three additional intermediate stages (Figure 1A) [4]. Time to reach anthesis from early bud stages is approximately 17–18 days. During this period, floral organ primordia sequentially emerge and differentiate, while the receptacle, the tissue that ultimately develops into the fleshy edible portion, undergoes coordinated expansion alongside the developing carpels [4,20,21].

### 2.2. Unique Developmental Features of Strawberry Flowers

Strawberry flowers exhibit distinctive developmental features distinct from most other angiosperms. This provides profound implications for both basic research and agricultural production. The most notable feature is the development of the “fruit” as a pseudocarp derived from the floral receptacle, with the true botanical fruits (achenes) embedded on its surface [22,23]. This accessory fruit structure arises from coordinated development between the receptacle and the numerous apocarpous (unfused) carpels that characterize the strawberry flower (Figure 1B) [21].

The strawberry flower typically produces numerous carpels, ranging from fewer than one hundred to more than three hundred depending on genotype, which are arranged on a conical receptacle [21]. Each carpel contains a single ovule; following fertilization, the ovule develops into a seed while the ovary wall develops into an achene, and the receptacle undergoes dramatic cell expansion and accumulation of sugars, pigments, and aromatic compounds [23,24]. Fruit growth and shape depend critically upon the position and number of achenes on the receptacle, and therefore upon the number of carpels in the flower. This dependency reflects the hormonal signals, particularly auxin, that achenes produce following fertilization, which in turn drive receptacle development [6]. Cytokinins also play a role in this process (detailed discussed in Section 4.2) [13].

Beyond their reproductive function, strawberry flowers also engage in biotic interactions that influence development. The synthesis of phenylpropenes, which are volatile compounds that function as floral attractants for pollinators, represents a dual-purpose adaptation, with these compounds also exhibiting antifungal activity [25]. Additionally, the flowers can exhibit compensatory responses to injury: simulated bud loss by the strawberry bud weevil (*Anthonomus signatus*) revealed that certain commercial cultivars can compensate for bud loss without reducing total yield [26]. However, harvest timing may be altered. This compensation mechanism likely involves hormonal reallocation, reflecting the remarkable developmental plasticity of the strawberry plant.

### 2.3. Developmental Differences Between Seasonal-Flowering and Perpetual-Flowering Strawberries

Strawberry cultivars (*F. vesca* and *F. × ananassa*) can be broadly categorized into seasonal-flowering (SF) and perpetual-flowering (PF, also known as everbearing) types based on their flowering habits [27,28]. These two types exhibit fundamentally different developmental trajectories that are primarily regulated by photoperiod response and vernalization requirements (Figure 1C). However, the terminology used to describe these habits has varied considerably across studies, and the physiological distinctions among the underlying types are not always clearly maintained. SF, also known as June-bearing or short-day, genotypes produce a single annual flowering flush in spring, induced by short days (SD) in autumn and typically requiring dormancy release (usually by vernalization) [18,29,30]. The chilling requirement for dormancy release varies widely among SF genotypes, ranging from approximately 100 to 1000 h below 7 °C, and is distinct from the process of floral induction, which occurs earlier under short-day conditions [27,29,31]. In these genotypes, the apical meristem differentiates into inflorescence structures during cool, short-day conditions [7]. The reproductive response is tightly coupled to seasonal environmental cues, ensuring that flowering occurs at an optimal time for pollination and fruit development [32]. Perpetual-flowering (PF) genotypes produce continuous flowering and fruiting throughout the growing season without a vernalization requirement [7]. The flowering pattern of PF genotypes typically takes the form of three or four successive phases: an autumn-initiated flowering phase, a flowering pause, and either a single stationary perpetual flowering phase or two perpetual flowering phases (the second being more intense) [33]. Within the PF category, two physiologically distinct subtypes can be recognized: everbearing (EB) types, which are responsive to long days (LDs) and flower under summer LD conditions; and day-neutral (DN) types, which are photoperiod-insensitive and flower under any day length, provided that temperatures are permissive [27,34]. In practice, however, “everbearing”, “day-neutral”, and “PF” have often been used interchangeably, and many cultivar descriptions do not distinguish between LD-responsive and photoperiod-insensitive phenotypes [7]. This terminological heterogeneity should be borne in mind when interpreting the literature.

Overall, the development of strawberry flowering is further displayed in Figure 1, and the comparison between SF and PF types is comprehensively listed in Table 1. These developmental differences between SF and PF strawberries are not merely of academic interest; they underpin the adaptation of cultivars to specific climates and production systems and represent key targets for breeding programs aimed at developing varieties with optimized flowering phenology for changing environmental conditions.

## 3. Environmental Regulation of Strawberry Floral Development

### 3.1. Regulation by Photoperiod

Photoperiod constitutes the primary environmental cue regulating floral transition in strawberry. SF varieties typically flower under short days (SD) while perpetual-flowering (PF) varieties flower under long days (LD) or are day-neutral [27,35,36]. The molecular framework underlying this differential response centers on the *CONSTANS (CO)–FLOW–RING LOCUS T 1* (*FT1*)–*TERMINAL FLOWER 1* (*TFL1*) regulatory module, which operates in both woodland (*F. vesca*) and cultivated (*F. × ananassa*) strawberries [7].

In plants, photoperiod perception is initiated via photoreceptors, particularly phytochromes and cryptochromes [7,37]. They undergo conformational changes in response to light [37,38]. This protein-encoded information is integrated with the circadian regulation of *CO* expression: *CO* is expressed on a rhythmic circadian cycle, with its expression peaking once every 24 h [39,40]. In the model plant *Arabidopsis* (*Arabidopsis thaliana*) under LD conditions, this peak occurs while photoreceptors are exposed to light, leading to post-transcriptional activation of *CO* [41,42]. Activated *CO* then triggers the induction of flowering integrator genes *FT* and *SUPPRESSOR OF CONSTANS 1* (*SOC1*) [43].

In *F. vesca*, the photoperiodic regulation of flowering operates through the *CO–FT1–SOC1–TFL1* module (the molecular architecture and SF/PF divergence are described in Section 5.1). Under LD conditions, the module represses flowering in SF genotypes, whereas in PF accessions lacking functional *FvTFL1*, the same photoperiodic signals promote flowering. This reversal of output relative to the *Arabidopsis* model is a defining feature of strawberry photoperiodism. In cultivated octoploid strawberry, a similar photoperiodic response has been observed: *FaCO* and *FaFT1* are upregulated in leaves under LD, correlating with *FaSOC1* and *FaTFL1* expression in crowns. Functional evidence for the role of *FaTFL1* as a floral repressor in cultivated strawberry is presented in Section 5.1.

### 3.2. Regulation by Temperature

Temperature acts as a critical modulator of strawberry flowering. It influences both independently and in interaction with photoperiod to regulate vernalization, meristem activity, and gene expression. In *F. vesca*, studies using the accession ‘Hawaii-4’ demonstrated that temperature-mediated changes in *FvTFL1* mRNA expression set critical temperature limits for photoperiodic flowering [44]. At temperatures below 13 °C, *FvTFL1* expression remains low under both SD and LD, allowing flower induction to occur independently of photoperiod. As temperature rises, *FvTFL1* mRNA levels gradually increase under LD; above 13 °C, SD becomes required for flower induction, which depends on the deactivation of *FvSOC1* and *FvTFL1*. Notably, an unknown transcriptional activator, functioning independently of *FvSOC1*, enhances *FvTFL1* expression at 23 °C, preventing photoperiodic flowering even under SD [44]. The expression pattern of *FvTFL1* across different temperature-photoperiod combinations corroborates its role as a thermal integrator. At 10 °C, *FvTFL1* mRNA levels are low in both photoperiods; under LD, a gradual increase in expression is detected with rising temperature. SD strongly suppresses *FvTFL1* at 16 °C, and a two-fold reduction in *FvTFL1* mRNA is also observed under SD at 13 °C. Intriguingly, under SD, *FvTFL1* mRNA levels gradually decrease with increasing temperature up to 16 °C, whereas several-fold activation occurs at the highest temperatures. Floral marker genes such as *FvFUL1* are clearly activated in flower-inductive SD treatments at temperatures of 10–16 °C compared with non-inductive conditions. Taken together, these data indicate that suppression of *FvTFL1* expression at cool temperatures allows flower induction to occur, whereas at intermediate temperatures SD is required for the *FvFT1/FvSOC1*-dependent reduction of *FvTFL1* mRNA; above the critical temperature limit, *FvTFL1* remains highly expressed even under SD, suppressing photoperiodic flowering.

In cultivated strawberry, studies with the cultivars ‘Tochiotome’ and ‘Akihime’ showed that the floral meristem is induced under SD and low temperature (SDLT) but not under LD and high temperature (LDHT) [45]. *FaFT1*, an orthologue of the *Arabidopsis* floral activator *FT*, is highly expressed in leaves under LDHT but not expressed under SDLT. By contrast, *FaTFL1* expression is higher under high temperature than under low temperature conditions, independent of daylength. As in *F. vesca*, floral inhibition pathways in *F. × ananassa* depend on *FaTFL1* regulation by daylength via *FaFT1* and by temperature [46].

Heide et al. examined floral induction in a range of latitudinal and altitudinal Norwegian populations of *F. vesca* under controlled environments and demonstrated a pronounced temperature × photoperiod interaction [47]. At 9 °C, flowers were initiated under both SD and LD conditions; at 15 °C and 18 °C, initiation occurred only under SD; and no initiation took place at 21 °C regardless of daylength. The critical photoperiod for SD floral induction was approximately 16 h and 14 h at 15 °C and 18 °C, respectively, with induction being incomplete at 18 °C [47]. Notably, a high-latitude population from 70°N (‘Alta’) exhibited an obligatory vernalization requirement, failing to flower even after 10 or 15 weeks of SD at 9 °C in the absence of vernalization, whereas other populations did not require vernalization. The expression of flowering-time genes again correlated with the flowering response: *FvFT1* was strongly suppressed in 14 h photoperiod compared with longer photoperiods, and parallel reductions in *FvSOC1* and *FvTFL1* expression were detected in the shoot apex [48,49]. These results are consistent with the model that the *FvFT1*–*FvSOC1*–*FvTFL1* pathway inhibits flower induction in photoperiods exceeding the critical limit.

### 3.3. Regulation by Light Quality

Beyond photoperiod (daylength), the spectral composition of light, particularly far-red (FR), blue (B), and red (R) light, exerts significant regulatory effects on strawberry flowering through the same *FvFT1/FvTFL1*-dependent molecular framework. In *F. vesca*, end-of-day FR and B light activate the expression of *FvFT1* and *FvSOC1* in both SD and LD strawberries, whereas low expression levels are detected under R light and SD treatments [50,51]. Via transgenic analysis, it is found that *FvFT1* advances flowering under FR and B treatments compared with R and SD treatments in the LD strawberry, and that *FvSOC1* is specifically required for the B light response. In the SD strawberry, however, flowering responses to these light quality treatments are reversed due to upregulation of the floral repressor *FvTFL1* in parallel with *FvFT1* and *FvSOC1*. These data highlight the central role of *FvFT1* in light quality-dependent flower induction in the LD strawberry and demonstrate that *FvTFL1* reverses not only photoperiodic requirements but also light quality effects on flower induction in the SD strawberry.

In a study using the day-neutral cultivar ‘Albion’, transplants were grown in a greenhouse under three light combinations with different far-red (FR) to blue (B) ratios—dominant blue light (FR:B = 1:5), dominant far-red light (FR:B = 5:1), and an equal ratio (FR:B = 1:1)—supplemented for long-day (LD; 24 h), short-day (SD; 10 h), or night-interruption (NI; 2 h) photoperiods using LEDs installed 70 cm above the plant canopy [52]. Under the LD photoperiod, dominant blue light (FR:B = 1:5) significantly increased flower bud induction compared with the other ratios. These findings identify FR:B = 1:5 as a potentially effective light treatment for improving flower bud induction in this cultivar under the specific conditions tested. However, these results are study-specific observations from a single cultivar under controlled greenhouse conditions; the extent to which they apply to other day-neutral cultivars, other production environments, and commercial cultivation systems requires further validation before being adopted as a general recommendation.

### 3.4. Integration of Environmental Signals

Strawberry plants integrate photoperiod, temperature, and light quality signals through overlapping molecular pathways, with *FvTFL1* serving as a central hub for this integration [44]. Photoperiod and temperature are major environmental signals affecting flowering in plants; although these pathways have been well characterized in the annual model *Arabidopsis*, much less is known in perennials. Many perennials, including woodland strawberry, are induced to flower in response to decreasing photoperiod and temperature in autumn and flower the following spring.

The integration mechanism operates through the coordinated regulation of *FvTFL1* expression. In contrast to *Arabidopsis*, the photoperiodic induction of flowering in strawberry occurs in SD, when the decrease in *FvFT1* and *FvSOC1* expression leads to lower mRNA levels of the floral repressor *FvTFL1*. Temperature modulates this photoperiodic response by setting critical limits for *FvTFL1* expression. The observed effect of photoperiod × temperature interaction on *FvTFL1* mRNA expression may allow strawberry to induce flowers at the correct time in different climates.

The critical photoperiod for flowering shifts with temperature. The *FvFT1–FvSOC1–FvTFL1* pathway inhibits flower induction in photoperiods longer than the critical limit (between 14 and 16 h at 18 °C in strawberry). However, at temperatures below 13 °C, this photoperiodic requirement is overridden, allowing flowering under both SD and LD. Above the critical temperature limit, *FvTFL1* is highly expressed even under SD, suppressing photoperiodic flowering entirely. This thermal modulation of photoperiodic response ensures that strawberry plants flower only when environmental conditions are favorable for reproductive success, integrating both seasonal daylength and temperature cues into a single developmental decision.

In summary, strawberry floral development is regulated by a sophisticated environmental signaling network in which photoperiod provides the primary inductive cue, temperature sets the permissive range for photoperiodic response, and light quality fine-tunes the output through the same *FvFT1/FvTFL1*-dependent pathway. The integration of these signals at the level of *FvTFL1* expression enables strawberry plants to synchronize flowering with seasonal conditions.

## 4. Hormonal Regulation of Strawberry Floral Development

### 4.1. Gibberellins

Gibberellins (GAs) constitute a pivotal class of phytohormones that orchestrate the balance between vegetative and reproductive growth in strawberry. Their regulatory effects exhibit a marked duality depending on concentration, developmental stage, and genetic background. In cultivated strawberry (*F. × ananassa*), exogenous application of GA_3_ has been shown to delay floral transition through the coordinated modulation of photoperiod signaling and nitrogen metabolism [14]. Weekly foliar application of GA_3_ to the short-day cultivar ‘Ninglu’ during the vegetative growth stage increased plant height by 57.7% and mean petiole length by 69.4%, while simultaneously enhancing leaf lamina expansion and vascular tissue development. Developmental analysis of the shoot apical meristem (SAM) revealed that although all control plants initiated flower buds within 40 days, only 77% of GA_3_-treated plants did so, confirming the inhibitory effect of GA on floral transition. At the molecular level, GA_3_ significantly upregulates the expression of photoperiod-related genes in leaves, including *GI*, *CO*, and *FT1*, which in turn activate floral integrators such as *SOC1* and *TFL1*. This activation cascade subsequently suppresses key floral activators, including *AP1*, *FUL*, and *LFY*, in the SAM, thereby delaying the transition from vegetative to reproductive development. Moreover, GA_3_ influences nitrogen partitioning in leaves by modulating the expression of genes associated with nitrogen metabolism (*NR*, *NIR*, *GS*, and *GOGAT*), transport (*NRT1.2/NRT1.7* and *AMT1.1*), and flowering time control (*FNR1* and *CRY1*) [14]. These findings collectively suggest that GA-mediated delay of floral transition operates through an integrated network that coordinates vegetative growth promotion, photoperiod signaling modulation, and nitrogen metabolic reprogramming.

The GA signaling pathway also interacts with other hormonal systems to regulate flowering. In woodland strawberry (*F. vesca*), overexpression of apple *TEMPRANILLO* (*MdTEM*) genes has been shown to downregulate *FvFT1*, *FvGA3OX1*, and *FvGA3OX2*, implicating GA biosynthesis genes in the flowering regulatory network [53]. Furthermore, there exists a well-documented trade-off between photoperiod-controlled flowering and GA signaling pathway-mediated runnering. In the seasonal-flowering cultivar ‘Sulhyang’, blue light treatment under non-runnering short-day conditions stimulated runner formation in association with the upregulation of *FaGRAS32* and *FaGA20ox4*, two genes in the GA pathway [35]. Independently, genetic analysis in *F. vesca* demonstrated that a deletion in *FveGA20ox4* abolishes runner formation, and this phenotype can be rescued by exogenous GA application [54]. The natural mutation in *FveGA20ox4* generates a phenotype that can be reversed by mutating *FveRGA1* (*REPRESSOR OF GIBBERELLIC ACID1*). This indicates the central role of GA signaling in determining whether an axial meristem produces a stolon or an inflorescence-bearing branch crown.

### 4.2. Cytokinins

Cytokinins (CKs) play a fundamental role in promoting cell division and meristem activity, thereby influencing both floral initiation and subsequent flower development in strawberry [13]. Detailed analyses of cytokinin dynamics during strawberry flower and fruit development have revealed a high concentration of trans-zeatin in flower buds and at anthesis, which subsequently decreases as the fruit approaches maturity. This temporal pattern suggests that CKs are particularly active during the early stages of floral development and receptacle initiation. Consistent with this observation, the spatio-temporal expression pattern of selected cytokinin oxidase/dehydrogenase (*CKX*) genes, which catalyze CK degradation, has been detected in receptacles at pre-anthesis stages.

The regulatory function of CKs extends beyond flower development. The transcription factor FveMYB117a has been identified as a key repressor of crown outgrowth, functioning by inhibiting CK accumulation [55]. This finding provides a mechanistic basis for understanding how CK homeostasis regulates the transition of axillary bud fate in this herbaceous perennial plant. Additionally, overexpression of the tRNA-type isopentenyl transferase gene *FveIPT2*, a key enzyme in CK biosynthesis, has been shown to increase anthocyanin and terpenoid accumulation in strawberry fruits with minimal impact on overall plant growth and development [56].

The interplay between CKs and auxin is particularly noteworthy in the context of floral development. *FveMYB117a* has been shown to control leaf morphology by coordinating auxin and cytokinin signals [55]. This indicates that the balance between these two hormone classes is critical for proper organ development. This hormonal coordination likely extends to the floral meristem, where CKs and auxin jointly regulate cell proliferation and differentiation during flower organogenesis.

### 4.3. Auxin, Abscisic Acid, and Ethylene

Auxin has emerged as a key regulator of flowering in strawberry, operating through a distinct pathway that converges on the activation of floral meristem identity genes. In cultivated strawberry, the expression of *AUXIN RESPONSE FACTOR4* (*FaARF4*) is significantly higher in the flowering stage than in the vegetative stage [57]. Overexpression of *FaARF4* in *Arabidopsis thaliana* and woodland strawberry resulted in transgenic plants flowering earlier than controls, whereas *FveARF4*-silenced strawberry plants exhibited delayed flowering. Mechanistically, ARF4 binds directly to the promoters of the floral meristem identity genes *APETALA1* (*AP1*) and *FRUITFULL* (*FUL*), inducing their expression and thereby promoting flowering.

The role of auxin in strawberry floral development is further underscored by studies on the *bare receptacle* (*bre*) mutant of woodland strawberry, which produces flowers with greatly reduced carpels and other floral organs [5]. The AP2 transcription factor BARE RECEPTACLE has been shown to regulate floral organogenesis via auxin pathways. This indicates that auxin signaling is essential not only for the initiation of flowering but also for the proper formation of floral organs. Additionally, the SAUR (SMALL AUXIN UP RNA) gene family members in woodland strawberry have been identified. Their expression profiles in response to auxin signals suggest that these genes may mediate multiple hormonal and environmental signals during flower development [58].

Abscisic acid (ABA) plays a complex role in strawberry floral development. In seasonal-flowering cultivars ‘Darselect’ and ‘All Star’, dormancy induction under short-day conditions at 12–15 °C was accompanied by increased ABA content and upregulation of the ABA biosynthesis gene *FaNCED1*, although the causal role of *FaNCED1* in dormancy induction remains to be functionally validated [59]. However, ABA also promotes fruit ripening by upregulating anthocyanin biosynthesis genes [60]. This indicates that its functions extend beyond dormancy into reproductive development. The antagonistic relationship between ABA and auxins is particularly evident in the regulation of postharvest physiology, yet both hormones play pivotal roles in regulating floral development, fruit yield, and quality attributes [61].

Ethylene is primarily associated with flower senescence and fruit ripening in strawberry. Ethylene application accelerates petal abscission in cultivated strawberry by upregulating ethylene response factors (ERFs) [62]. In contrast, the role of ethylene in floral induction is less clearly defined. Transcriptomic profiling of floral induction in the cultivated strawberry cultivar ‘Benihoppe’ revealed that *EBF1-like* was upregulated at the early stage of floral induction, while *EIN3-like1* and *ERF1B* were downregulated at 15 weeks [12]. This suggests that ethylene signaling may negatively regulate floral induction in this genotype. However, this evidence is correlative and based on transcript abundance rather than functional analysis, and it is derived from a single cultivar under specific experimental conditions. The proposal that ethylene promotes floral induction in some perpetual-flowering varieties by repressing *TFL1* [10,63] is not supported by direct experimental evidence in strawberry and should be considered speculative.

### 4.4. Integration of Hormonal Signals

The regulation of strawberry floral development does not rely on any single hormone acting in isolation; rather, it emerges from a complex network of hormonal interactions that integrate endogenous developmental cues with environmental signals.

#### 4.4.1. GA and Cytokinin

GA and CK exert opposing effects on strawberry floral development. GA application delays floral transition by promoting vegetative growth. This leads to increased plant height and petiole length and a reduced proportion of plants initiating flower buds. CK is associated with the early stages of floral development and receptacle initiation. The concentration of trans-zeatin is high in flower buds and at anthesis, and declines as fruit approaches maturity. This temporal pattern indicates that CKs are particularly active during early reproductive development, while GA favors vegetative growth. The balance between these two hormones therefore appears to determine whether the shoot apical meristem remains in a vegetative or reproductive state. The physiological basis of this antagonism has been supported by combined analysis of endogenous hormone dynamics and gene expression during floral induction.

#### 4.4.2. GA/Auxin and ABA

The antagonistic relationship between GA/auxin and abscisic acid (ABA) is particularly relevant in dormancy and flowering. In SF strawberry varieties, ABA is primarily associated with the induction of dormancy under SD conditions, whereas GA application breaks dormancy. This reciprocal relationship ensures that the plant integrates promotive and inhibitory signals appropriately: when conditions favor vegetative growth, GA and auxin levels are high, and ABA accumulation is suppressed; when conditions favor dormancy or developmental arrest, ABA levels rise and GA signaling is attenuated. The physiological consequence of this antagonism is that strawberry plants coordinate the timing of dormancy release with the onset of favorable conditions for flowering and fruit development.

#### 4.4.3. Auxin and CK

The interplay between auxin and CK is critical for proper organ development, including the floral meristem. The balance between these two hormone classes determines axillary bud fate, with relatively higher auxin activity associated with dormant buds and relatively higher CK activity associated with non-dormant buds. Exogenous cytokinin application can trigger cell fate conversion and promote reproductive development. This coordination likely extends to the floral meristem, where CK and auxin jointly regulate cell proliferation and differentiation during flower organogenesis. The physiological significance of this interaction is that it allows the plant to integrate internal developmental signals with external environmental cues to determine whether an axillary meristem produces a runner, a branch crown, or an inflorescence.

#### 4.4.4. Ethylene and Auxin

Ethylene is primarily associated with flower senescence and fruit ripening in strawberry. Ethylene application accelerates petal abscission, indicating its role in promoting senescence-related processes. However, ethylene also appears to influence floral induction, with transcriptomic evidence suggesting that ethylene signaling negatively regulates floral induction during the early stages. The crosstalk between ethylene and auxin is particularly relevant because ethylene can modulate auxin signaling, and auxin can influence ethylene biosynthesis. This interaction ensures that senescence-related signals are integrated with developmental programming, allowing the plant to coordinate reproductive development with the physiological status of the whole plant. In summary, strawberry floral development is regulated by a sophisticated hormonal network (Figure S1). The integration of these hormonal signals ensures that strawberry plants coordinate reproductive development with endogenous physiological status and environmental conditions, ultimately optimizing the timing and success of flowering.

## 5. Molecular Networks Integrating Hormonal, Environmental and Genetic Cues

### 5.1. Core Modules in the Photoperiodic Pathway

The photoperiodic flowering module in strawberry is orchestrated around a *CO-FT1-SOC1-TFL1* regulatory cascade that operates in both woodland and cultivated strawberries [7]. In general terms, photoperiod perception is initiated by phytochromes and cryptochromes. This protein-encoded information is integrated with the circadian regulation of *CONSTANS* (*CO*) expression. In *F. vesca*, the output of this conserved module is reversed relative to the *Arabidopsis* model. In SF *F. vesca*, the LD-activated *FvFT1–FvSOC1–FvTFL1* pathway represses flowering, because *FvSOC1* upregulates the floral repressor *FvTFL1* [64]. By contrast, in PF accessions that lack functional *FvTFL1*, overexpression of *FvCO* induces *FvFT1* expression in leaves. This correlates with upregulation of *FvSOC1* and floral meristem identity genes such as *FvAP1* at the shoot apical meristem (SAM), thereby promoting flowering. At the protein level (in *Arabidopsis*), FT and TFL1 interact with the bZIP transcription factor FD through 14–3–3 proteins [65]. FT interactions with FD via 14–3–3 form a transcriptional activator complex for floral identity genes that promote flowering. TFL1 competing with FT for FD interaction leads to a repressor complex that inhibits flowering and promotes vegetative growth. This competitive interaction demonstrated in *Arabidopsis* provides a referential molecular mechanism through which the relative abundance of FT and TFL1 may influence the developmental fate of the SAM in strawberry. However, direct protein–protein interaction evidence in *Fragaria* is currently lacking.

In cultivated octoploid strawberry (*F. × ananassa*), functional characterization of the core photoperiodic module has revealed both conserved and divergent features. Overexpression of *FaSOC1* in the seasonal-flowering cultivar ‘Camarosa’ significantly repressed expression of *FaFT2* and *FaFT3*. The expression of floral meristem identity genes *FaLFYa*, *FaAP1*, and *FaFUL* in the crown also shows downregulation, while the expression of *FaTFL1* shows upregulation [64]. Transgenic silencing of *FaTFL1* in the short-day cultivar ‘Elsanta’ has been shown to induce perpetual flowering under long days without direct changes in vegetative reproduction, indicating that *FaTFL1* encodes a major floral repressor in cultivated strawberry [66]. Nevertheless, the regulation of *FaTFL1* varies widely from cultivar to cultivar and is correlated with floral induction. This indicates that the transcription of *FaTFL1* occurs at least partially independently of the *FaFT1–FaSOC1* module.

Additional regulatory layers in the octoploid context are beginning to emerge. The B-box transcription factor (BBX) family has been identified as a potentially important component: 51 *FaBBX*s have been identified from cultivated strawberry and 16 *FvBBX*s from diploid woodland strawberry, revealing significant expansion of this gene family in the octoploid genome [67]. Functional characterization of *FaBBX28c1* suggests that it may act as a negative regulator of flowering in cultivated strawberry. The functions of other BBXs in flowering time regulation in *F. × ananassa* remain largely uncharacterized.

### 5.2. Key Genes in Floral Meristem Identity and Organogenesis

Downstream of the photoperiodic module, a suite of floral meristem identity genes orchestrates the transition from inflorescence meristem to floral meristem and the subsequent specification of floral organ identity. In *F. vesca*, the *FveFT2* florigen/*FveTFL1* antiflorigen balance is critical for the control of seasonal flowering in strawberry, while *FveFT3* modulates axillary meristem fate and yield [10]. The floral meristem identity gene *LEAFY* (*LFY*) plays an important role in the activation of downstream floral organ identity genes [68]. In woodland strawberry, four *LEAFY* homologues have been identified, with *FveLFYa* being essential for floral patterning. In the *fvelfya* mutant, downregulation of B-class genes *FveAP3a, FveAP3b*, *FvePIa*, and *FvePIb* has been observed. This is consistent with the conserved role of LFY in activating B-class gene expression for petal and stamen identity specification. Notably, *FveAP1* expression was not altered in *fvelfya* compared to wild type.

The AP2 transcription factor BARE RECEPTACLE (BRE) has been identified as essential for floral organogenesis, particularly carpel initiation, acting through the auxin pathway [5]. The *bre* mutant produces flowers with greatly reduced carpels and other floral organs. Additionally, *FveSEP3*, a *MADS-box* gene, plays essential roles in flower organogenesis, with mutants showing conversion of petals, stamens, and carpels to sepaloid organs [69]. In situ hybridization with the *F. vesca AGAMOUS* homolog, *FvAG*, has revealed expression in young stamen and carpel primordia, indicating the conservation of floral regulatory genes across Rosaceae [20]. In cultivated strawberry, CRISPR/Cas9-mediated mutagenesis of *FaTM6*, a B-class *MADS-box* gene, resulted in abnormal anther development. Ectopic expression of the loquat *LEAFY* homolog *EjLFY-1* in *F. × ananassa* accelerated flowering and reduced the time to flower induction, while maintaining normal floral and fruit morphology in the first generation [70].

Transcriptomic analyses of *F. vesca* flower development have identified coexpression networks linked to floral organogenesis, which reveal stage-specific gene expression patterns across different tissues [21]. These networks, visualized via the eFP browser, highlight the dynamic regulation of gene expression throughout floral differentiation. Moreover, gene expression profiling of leaves in conjunction with floral bud differentiation in cultivated strawberry ‘Akihime’ has identified genes whose expression in leaves changes synchronously with the transition from SAM to FM, offering potential biomarkers for floral initiation [71].

It should be emphasized that functional characterization of floral meristem identity genes in the octoploid context faces unique challenges arising from the complex polyploid genome and high heterozygosity of *F. × ananassa*. The phenomenon of subgenome dominance inherent to polyploid species means that the four subgenomes may contribute differentially to flowering regulation [72]. Furthermore, the perpetual flowering trait in octoploid strawberry appears to have a different genetic basis than in *F. vesca*: while the lack of functional *FvTFL1* underlies perpetual flowering in diploid *F. vesca*, the genetic determinants of perpetual flowering in octoploid cultivars such as ‘Albion’ and ‘Seascape’ are less clearly defined and may involve additional loci [73]. Key genes discussed in this subsection are listed in Table 2.

### 5.3. Integration of Hormone Signals into the Molecular Network

The molecular network regulating strawberry flowering is not a simple linear cascade but rather a highly integrated system in which hormone signaling pathways converge on the core photoperiodic module (Figure 2). Transcriptomic analyses have provided substantial evidence for this integration. In a time-course transcriptomic profiling study of floral induction in cultivated strawberry, the most enriched KEGG pathway among differentially expressed genes was plant hormone signal transduction, with key genes in the auxin signaling pathway, including *AUX1*, *TIR1-like*, *IAA20-like*, and *SAUR38* [12]. These genes are significantly upregulated during the transition from vegetative to reproductive stage. This broad activation of hormone signaling components during floral induction underscores the key participation of hormonal integration in the flowering decision. Furthermore, transcriptomic analysis of seasonal flowering genotypes of cultivated strawberry under natural conditions has identified genes associated with the photoperiodic pathway, including *TFL1* and *SOC1*, as well as several linked to phytohormone regulation such as *XTH23*, *PP2*, and *EIN3* [15]. This suggests crosstalk between photoperiod and hormone signaling during floral initiation.

GA application delays floral transition by upregulating *GI*, *CO*, and *FT1*, which activate *SOC1* and *TFL1*, subsequently suppressing *AP1*, *FUL*, and *LFY* in the SAM [14]. Combined analysis of *DELLA* expression with cytokinin signaling components indicates that GA and cytokinin may act antagonistically on floral induction [12]. Cytokinin signaling appears to function primarily during the reproductive phase, with receptor genes *CRE1* and *AHP* upregulated at 15 weeks [12]. Auxin signaling integrates into the floral regulatory network through the activation of floral meristem identity genes. Auxin integrates into this network by directly activating floral meristem identity genes. FaARF4 is significantly upregulated during flowering and binds directly to the promoters of *AP1* and *FUL*, inducing their expression and promoting flowering [57]. Transcriptomic profiling of floral induction in the cultivated strawberry cultivar ‘Benihoppe’ revealed that *EBF1-like* was upregulated at the early stage while *EIN3-like1* and *ERF1B* decreased at 15 weeks [12]. These expression changes are consistent with a possible negative role for ethylene signaling during floral induction, but they are correlative and do not by themselves establish a causal relationship between ethylene signaling and the flowering response. JA signaling may also play a negative role, with MYC2 significantly downregulated during floral induction [12]. ABA signaling participates in the early stages, with ABA receptor genes *PYL/PYR* upregulated at 13 weeks while PP2C is decreased [12].

## 6. Discussion and Future Perspectives

### 6.1. Synthesis of the Integrated Regulatory Network

The evidence synthesized in this review consolidates a multilayered model of strawberry floral development. This model can be conceptualized as a hierarchy in which photoperiod and temperature provide the primary inductive and permissive cues, respectively. Phytohormones, including GA, CKs, auxin, ABA, and ethylene, serve as endogenous transducers that modulate the core photoperiodic module at multiple nodes. The floral meristem identity genes *LFY*, *AP1*, *FUL*, *SEP3*, and *TM6* then execute the developmental program of floral organogenesis.

Despite this progress, several critical gaps remain. The molecular mechanism of temperature sensing and signal transduction in strawberry flowering, particularly the identification of the thermosensor and the pathway through which temperature modulates *FvTFL1* expression, is still not fully understood. In *F. vesca*, the temperature-dependent regulation of *FvTFL1* has been dissected at the physiological and transcriptional levels [44], yet the upstream components that translate temperature into changes in *FvTFL1* transcription remain unidentified. Furthermore, the integration of light quality signals into the core flowering network is only beginning to be understood. Although the recent discovery of the FvCRY2–FvbHLH78 module provides a concrete molecular link between blue-light perception and floral gene activation [74], he broader network of light-responsive regulators and their interactions with the core photoperiodic module remain to be fully characterized (see Section 6.4). Otherwise, the role of epigenetic regulation, including histone modifications, DNA methylation, and non-coding RNAs, in modulating the expression of key floral regulators such as *FvTFL1* across different environments and developmental stages is largely unexplored in strawberry. Addressing these gaps will require the integration of genetic, genomic, and physiological approaches in a manner that captures the dynamic and context-dependent nature of the flowering regulatory network.

### 6.2. From Diploid Model to Octoploid Crop: Bridging the Knowledge Gap

Studies in the diploid woodland strawberry *F. vesca* have established an indispensable framework for understanding the core regulatory logic of flowering. However, the transfer of this knowledge to the cultivated octoploid strawberry is constrained by several fundamental differences. The most significant of these is the observation that the regulation of *FaTFL1* varies widely from cultivar to cultivar and occurs at least partially independently of the *FaFT1–FaSOC1* module. This finding challenges the assumption that the *F. vesca*-derived model can be directly extrapolated to the octoploid context and underscores the necessity for cultivar-specific analyses. Transgenic silencing of *FaTFL1* in the short-day cultivar ‘Elsanta’ is sufficient to induce perpetual flowering under long days [66], confirming that FaTFL1 encodes a major floral repressor in cultivated strawberry; however, the upstream regulators that control *FaTFL1* transcription in different cultivars remain largely unknown.

The complexity of the octoploid genome, characterized by four subgenomes, high heterozygosity, and potential subgenome dominance, introduces additional layers of regulatory complexity that cannot be predicted from the diploid model alone. The perpetual flowering trait in octoploid cultivars such as ‘Albion’ and ‘Seascape’ appears to have a different genetic basis than in *F. vesca*: while the loss-of-function mutation in *FvTFL1* underlies perpetual flowering in diploid woodland strawberry [63], the genetic determinants of perpetual flowering in octoploid cultivars are less clearly defined and may involve additional loci that have yet to be identified.

These considerations lead to the conclusion that while the *F. vesca* model provides essential conceptual and molecular anchors, it must be used as a starting point rather than an endpoint. Future research should prioritize the functional characterization of flowering regulators in octoploid strawberry on a cultivar-specific basis. Forward genetics and comparative genomics can be performed for the identification of octoploid-specific regulatory components. The development of a unified but adaptable model is necessary and promising, which accounts for both shared and divergent features between diploid and octoploid Fragaria species.

### 6.3. Hormonal Research Avenues in Strawberry Flowering

Future research on hormonal regulation of strawberry flowering should address several unexplored areas that hold promise for both fundamental understanding and practical application. The role of brassinosteroids (BRs) in strawberry flowering remains largely unstudied despite evidence from *Arabidopsis* that BRs promote flowering by upregulating *FT* [75]. The identification of a potential auxin–BR regulatory module in early receptacle elongation suggests that BRs may participate in the hormonal network governing strawberry floral and receptacle development. However, the specific functions of BR signaling in floral induction and organogenesis require systematic investigation. Similarly, the interaction between strigolactones (SLs) and CKs in regulating axillary bud differentiation is critical for determining whether an axillary meristem produces a runner or an inflorescence. The recent identification of *FveMYB117a* as a key repressor of crown outgrowth through inhibition of cytokinin accumulation provides a starting point for dissecting the hormonal control of axillary meristem fate, but the roles of SLs and their interactions with CKs and auxin in this process remain to be defined.

Another promising avenue is the study of hormone transporters. The distribution of auxin during floral meristem initiation and organogenesis is likely mediated by specific PIN-FORMED (PIN) efflux carriers, yet the identities and functions of strawberry PIN homologs in floral tissues have not been systematically characterized. Given that auxin plays a central role in activating floral meristem identity genes through ARF4-mediated transcriptional regulation of *AP1* and *FUL* [57], understanding how auxin is transported to and distributed within the floral meristem is essential for a complete mechanistic picture. Furthermore, the role of small RNAs in hormonal regulation of flowering warrants investigation. The characterization of miRNA–hormone networks could provide new insights into post-transcriptional regulation of flowering, and the identification of miRNAs that target hormone signaling components.

### 6.4. Light-Signal Research Avenues and Environmental Integration

Although the role of photoperiod in strawberry flowering has been extensively studied, the perception, transduction, and integration of other light parameters remain poorly understood. Most current knowledge on light quality comes from controlled-environment studies focusing on specific far-red (FR) to blue (B) ratios, such as the FR:B = 1:5 treatment that enhanced flower bud induction in the day-neutral cultivar ‘Albion’ [52]. However, how these light signals are perceived and integrated at the molecular level is only beginning to be elucidated. The recent identification of the FvCRY2–FvbHLH78 module, which directly binds and activates the promoters of *FvFT*, *FvAGL42*, *FvLFY*, and *FvSEP3* in *F. vesca* [74], provides the first concrete molecular link between blue-light perception and floral gene activation in strawberry. Yet it remains unknown how this module interacts with the core photoperiodic *CO–FT1–SOC1–TFL1* pathway and whether phytochromes (e.g., PHYA and PHYB) mediate the far-red and red light responses through distinct or shared downstream components.

Furthermore, the interaction between light and temperature at the molecular level is largely unexplored. While physiological studies have shown that temperature sets critical limits for photoperiodic flowering and that low temperatures can override photoperiodic requirements [44,47], the specific photoreceptor-thermosensor crosstalk remains undefined. Future research should prioritize the functional characterization of other photoreceptors (such as UV B receptors and phytochromes) in *F. vesca* and *F. × ananassa*, and the identification of their downstream targets using transcriptomic, proteomic, and chromatin accessibility approaches. Additionally, from an applied perspective, optimizing light recipes for controlled-environment agriculture (CEA) requires a deeper understanding of how light intensity (e.g., daily light integral) and dynamic spectral changes interact with temperature and cultivar-specific genetic backgrounds to determine flowering time and fruit quality. This systems-level understanding will be essential for designing precision lighting strategies that maximize yield and resource use efficiency in protected strawberry cultivation.

### 6.5. Genetic Innovation and Next-Generation Strawberry Varieties

The convergence of advances in genomics, transcriptomics, and genome editing technologies creates opportunities for the genetic improvement of strawberry flowering traits. Multi-omics approaches, integrating transcriptomics, metabolomics, and proteomics, have begun to reveal the complex regulatory networks underlying floral development in strawberry. An integrated multi-omics strategy combining transcriptome, sRNAome, and degradome sequencing has been applied to analyze petal coloration during flower development in pink-flowered strawberry. This analysis identifies 317 differentially expressed miRNAs and 2134 target genes enriched in transcriptional regulation pathways [76]. Extending this approach to the analysis of floral initiation and organogenesis could reveal novel regulatory components and provide a systems-level understanding of the flowering process.

The development of single-cell and spatial transcriptomics technologies represents a particularly exciting frontier for strawberry flowering research. These technologies enable the profiling of gene expression at cellular resolution within complex tissues. This is promising for revealing the spatiotemporal dynamics of floral meristem organization and the cell-type-specific expression patterns of key regulators.

Finally, the development of parthenocarpic strawberries, varieties that produce fruit without fertilization, represents a potentially transformative innovation for strawberry production. Parthenocarpy could increase yield, reduce dependency on pollinators (whose populations are declining globally), and ensure fruit set under environmental conditions that impair pollination. Genetic manipulation of auxin or GA signaling pathways could induce parthenocarpy, and the growing understanding of the hormonal regulation of receptacle development provides a foundation for targeted approaches. However, the introduction of parthenocarpy must be carefully evaluated for its effects on fruit quality, seed production, and the ecological interactions that depend on pollen and nectar resources.

## 7. Conclusions

Strawberry flower formation is regulated by a multilayered regulatory network. This review has synthesized the morphological framework of floral differentiation, the environmental cues (photoperiod, temperature, and light quality) that govern floral induction, the hormonal pathways that transduce these signals, and the molecular networks that integrate them. The *CO–FT1–SOC1–TFL1* module emerges as the conserved core pathway of photoperiodic regulation in *Fragaria*, with *FvTFL1* functioning as the central hub integrating photoperiodic and thermal signals (accessions ‘Hawaii-4’ and seasonal-flowering Norwegian accessions). The competitive interaction between FT–FD–14-3-3 activator and TFL1–FD–14-3-3 repressor complexes determines floral fate at the shoot apical meristem (accessions *Arabidopsis*). Hormonal signals modulate this core module at multiple nodes. In *F. vesca*, the divergence between seasonal-flowering and perpetual-flowering strategies is principally attributable to the presence or absence of functional *FvTFL1*. In cultivated octoploid strawberry, however, the genetic architecture of the perpetual-flowering trait is more complex: *FaTFL1* regulation is cultivar-dependent and may occur partly independently of the *FaFT1*–*FaSOC1* module.

Despite these advances, critical gaps remain, particularly in understanding the molecular basis of temperature sensing, the functions of octoploid-specific regulators, and the epigenetic and post-transcriptional layers of flowering control. Addressing these gaps will require cultivar-specific functional characterization that bridges the diploid model and the octoploid crop. This integrated framework provides both a conceptual foundation for future research and a practical reference for breeding strawberry varieties with optimized flowering phenology.

## Figures and Tables

**Figure 1:**
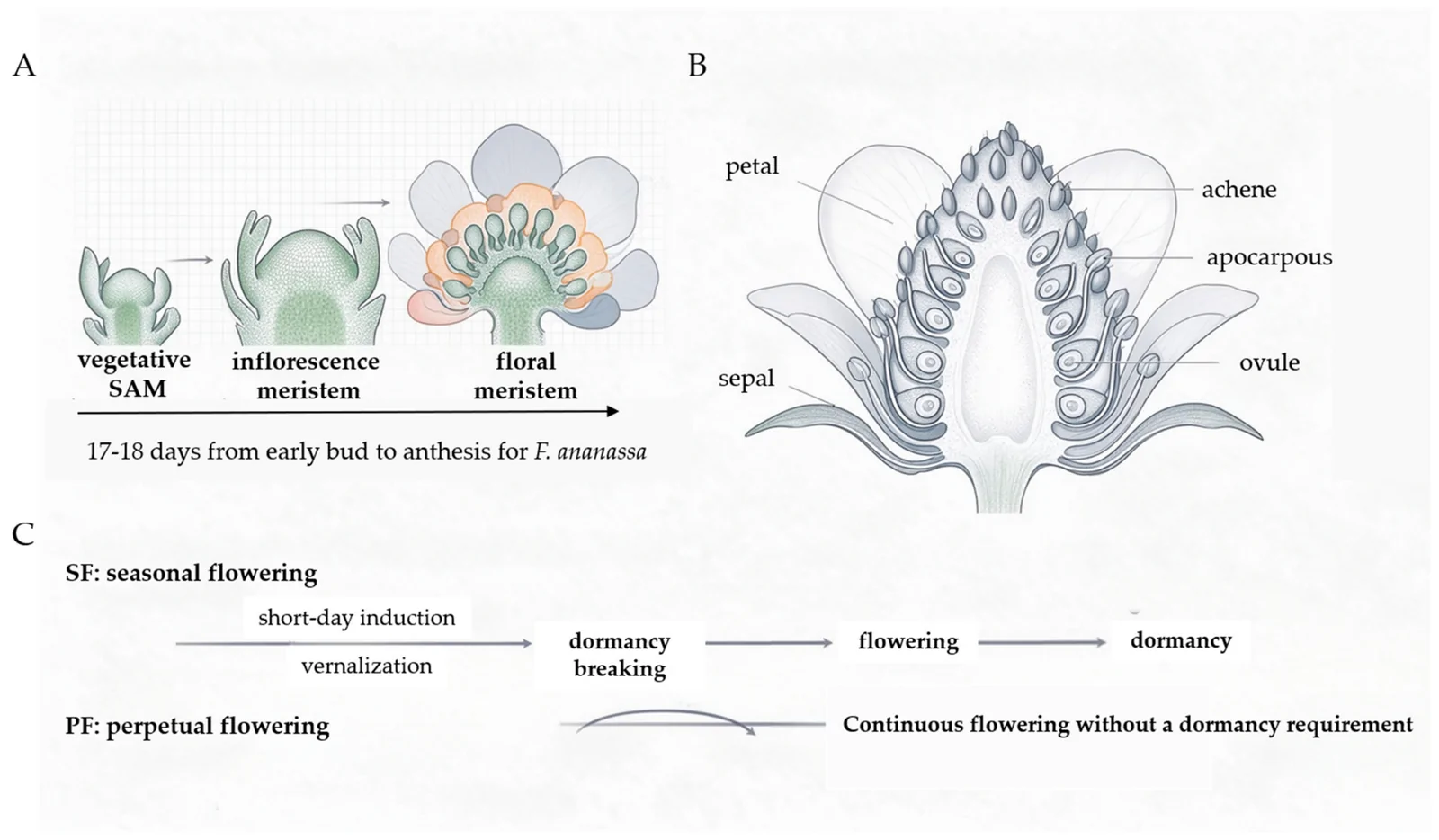
Morphological and developmental framework of strawberry flower formation. (**A**) Schematic representation of the morphogenetic progression of floral differentiation in strawberry. The three meristem stages, vegetative SAM, inflorescence meristem, and floral meristem, are shown with their characteristic morphological transitions. The timeline (bottom) indicates the approximate duration from early bud stages to anthesis (17–18 days for *F. × ananassa*). (**B**) Longitudinal section of a strawberry flower highlighting its unique structural features, including the pseudocarpic nature of the fruit, the apocarpous (unfused) carpels arranged on a conical receptacle, and the single ovule contained within each carpel. Labels in the panel refer to structures at two developmental stages: ovule and ovary indicate the pre-fertilization stage, whereas achene and seed indicate the post-fertilization stage. Following fertilization, each ovule develops into a seed, and the ovary wall develops into an achene, while the receptacle develops into the fleshy edible portion. (**C**) Comparative developmental trajectories of seasonal-flowering (SF) and perpetual-flowering (PF) strawberry varieties.

**Figure 2:**
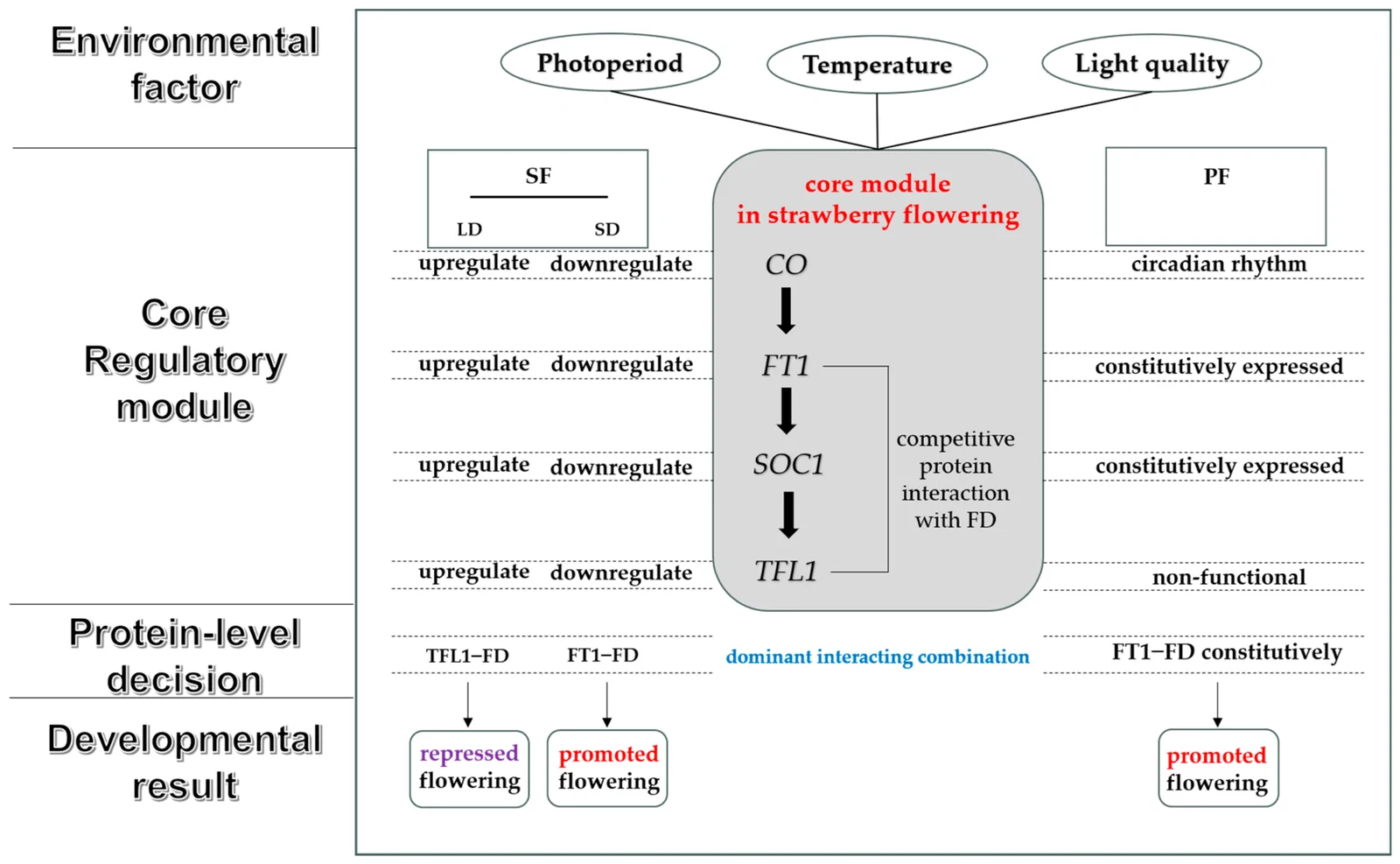
Integrated molecular network of strawberry floral development. SF indicates seasonal-flowering, and PF indicates perpetual-flowering. LD indicates long day, and SD indicates short day. Thick solid arrows indicate regulation. Thin line arrows indicate induction. (Top layer) Environmental inputs—photoperiod, temperature, and light quality—converge on the core photoperiodic module. Short days (SD) promote *FT1*/*SOC1* downregulation and *TFL1* suppression, whereas long days (LD) activate *FT1*/*SOC1* and upregulate *TFL1*; these photoperiodic responses were characterized in *F. vesca* (accessions ‘Hawaii-4’ and seasonal-flowering Norwegian accessions) and in *F. × ananassa* (cultivars ‘Camarosa’ and ‘Elsanta’). Low temperatures (<13 °C) suppress *TFL1* expression, overriding the photoperiodic requirement; high temperatures (>13 °C) enhance *TFL1* expression through both *FvSOC1*-dependent and *FvSOC1*-independent pathways; these temperature thresholds were established in *F. vesca* ‘Hawaii-4’. Far-red (FR) and blue (B) light activate *FT1*/*SOC1*, whereas red (R) light has little effect; these light-quality responses were characterized in *F. vesca*. (Center layer) The core cascade *CO → FT1 → SOC1 → TFL1* is conserved in both *F. vesca* and *F. × ananassa*. In seasonal-flowering (SF) *F. vesca* accessions, the LD-activated pathway upregulates *FvTFL1*, repressing flowering. In perpetual-flowering (PF) *F. vesca* accessions such as ‘Hawaii-4’ (right track), loss-of-function mutations in *FvTFL1* permit continuous flowering. In cultivated octoploid strawberry, *FaSOC1* overexpression in ‘Camarosa’ represses *FaFT2*/*FaFT3* and floral meristem identity genes while upregulating *FaTFL1*, and transgenic silencing of *FaTFL1* in ‘Elsanta’ induces perpetual flowering. (Bottom layer) At the protein level, FT and TFL1 compete for binding to FD via 14-3-3 proteins. This competitive interaction was demonstrated in *Arabidopsis* and is presumed to operate in strawberry, but direct protein–protein interaction evidence in *Fragaria* is currently lacking. The FT–FD–14-3-3 complex activates floral identity genes, whereas the TFL1–FD–14-3-3 complex represses them.

**Table 1:** Comparison of seasonal-flowering (SF) and perpetual-flowering (PF) strawberry varieties.

Category	Feature	SF	PF
environmental requirement	photoperiod response	SD-induced; LD represses flowering	Day-neutral or LD; continuous flowering under favorable conditions
	dormancy release requirement	yes	no
	optimal flowering temperature	*FvTFL1* expressed until vernalization	*FvTFL1* repressed constitutively or loss-of-function
developmental characteristics	flowering pattern	10–15 °C under SD	15–25 °C; less temperature-sensitive
	apical meristem fate	vegetative growth maintained under LD; reproductive transition occurs under SD and cool conditions	continuous reproductive transition under favorable conditions
representative cultivars and agricultural implication	representative cultivars	“Camarosa”, “Elsanta”, “Ninglu”	“Albion”, “Seascape”, some *F. vesca* accessions
	agricultural implication	single annual harvest; requires winter chilling or dormancy-breaking treatment	multiple harvests per season; suitable for controlled-environment production

Note: SF indicates seasonal-flowering and PF indicates perpetual-flowering. SD indicates short-day condition, and LD indicates long-day condition.

**Table 2:** Key genes and their functions in strawberry floral development.

Gene	Species	Function
*FvTFL1*	*F. vesca*	floral repressor; downregulation required for vernalization-induced flowering
*FaTFL1*	*F. × ananassa*	major floral repressor; cultivar-specific regulation
*FvFT1/FaFT1*	both	florigen; activated by CO under LD; moves to SAM
*FveFT2*	*F. vesca*	non-photoperiodic florigen; permits SD flowering
*FveFT3*	*F. vesca*	modulates axillary meristem fate; increases yield
*FvCO/FaCO*	both	photoperiodic regulator; rhythmic expression; activates *FT1*
*FvSOC1/FaSOC1*	both	floral integrator; upregulates *TFL1* in SF; promotes flowering in PF
*FveLFYα*	*F. vesca*	floral meristem identity; activates B-class genes
*FaARF4*	*F. × ananassa*	auxin response factor; directly activates *AP1* and *FUL*
*FveSEP3*	*F. vesca*	MADS-box; floral organ identity
*FaTM6*	*F. × ananassa*	B-class MADS-box; stamen identity
*BRE*	*F. vesca*	AP2 transcription factor; carpel initiation via auxin pathway
*FveMYB117a*	*F. vesca*	repressor of crown outgrowth; inhibits cytokinin accumulation
*FaBBX2* *8c1*	*F. × ananassa*	B-box transcription factor; negative regulator of flowering

## Data Availability

No new data were created or analyzed in this study. Data sharing is not applicable to this article.

## Supplementary Materials

The following supporting information can be downloaded at https://www.mdpi.com/article/10.3390/plants15213378/s1, Figure S1. Hormonal regulation network of strawberry floral development. Solid arrow (→) indicates positive regulation. Solid flat-bar line (⊣) indicates negative regulation. Dashed line indicates unverified regulation. Thin line under the hormones indicates an antagonistic relationship. Table S1: Evidence categories for major mechanistic claims in strawberry flowering.
