# Supplementary material for: Integrating Hormonal, Environmental, and Genetic Cues in Strawberry Flowering
Source: Plants (Basel). 2026 Nov 1;15(21):3378. doi: 10.3390/plants15213378 (PMC13766006; doi:10.3390/plants15213378)
Supplement: Supplementary file 1 [file plants-15-03378-s001.zip › plants-4599267-supplementary.pdf]

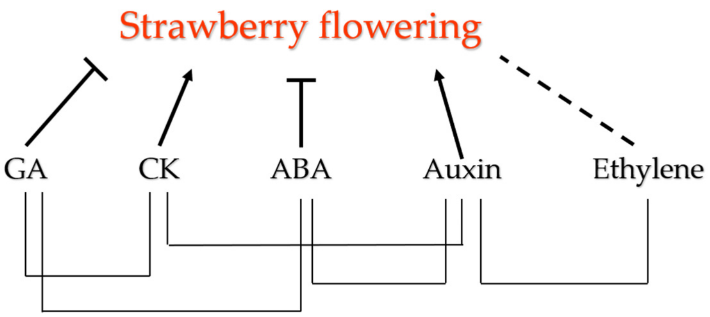

Figure S1. Hormonal regulation network of strawberry floral development. Solid arrow (→) indicates positive regulation. Solid flat-bar line (⊣) indicates negative regulation. Dashed line indicates unverified regulation. Thin line under the hormones indicates antagonistic relationship.

Table S1. Evidence categories for major mechanistic claims in strawberry flowering.

Regulatory component	*Fragaria vesca*	*Fragaria × ananassa*	Transcriptomic correlation	Heterologous (*Arabidopsis thaliana*)	Proposed/inferred
CO–FT1–SOC1–TFL1 cascade	✓	✓	✓		
TFL1 as floral repressor	✓	✓			
Temperature regulation of *FvTFL1*	✓				
FveFT2/FveFT3 functions	✓				
GA delays floral transition		✓	✓		
FveGA20ox4 controls runnering	✓				
Cytokinin in receptacle development	✓	✓	✓		
ARF4 activates *AP1/FUL*	✓			✓	

BRE regulates carpel initiation	✓				
FveSEP3 in floral organogenesis	✓				
FaTM6 in stamen identity		✓			
Ethylene represses *TFL1*			✓		✓
JA negatively regulates floral induction			✓		✓
DELLA integrates GA/CK signals			✓	✓	✓
FT–FD–14-3-3 competition				✓	✓

Note: "✓" icon indicates evidence available.
